# Electroactive poly(sulfobetaine-3,4-ethylenedioxythiophene) (PSBEDOT) with controllable antifouling and antimicrobial properties†
†Electronic supplementary information (ESI) available. See DOI: 10.1039/c5sc03887a


**DOI:** 10.1039/c5sc03887a

**Published:** 2015-12-18

**Authors:** Bin Cao, Chen-Jung Lee, Zhipeng Zeng, Fang Cheng, Fujian Xu, Hongbo Cong, Gang Cheng

**Affiliations:** a Department of Chemical and Biomolecular Engineering , University of Akron , Akron , Ohio 44325 , USA . Email: gc@uakron.edu ; http://gozips.uakron.edu/∼gc/index.html ; Email: hcong@uakron.edu ; Fax: +1-330-972-7250; b School of Pharmaceutical Engineering , Dalian University of Technology , Dalian , Liaoning Province 116024 , China; c Key Laboratory of Carbon Fiber and Functional Polymers (Beijing University of Chemical Technology) , Ministry of Education , Beijing Laboratory of Biomedical Materials , Beijing University of Chemical Technology , Beijing 100029 , China

## Abstract

This paper reports a multifunctional zwitterionic poly(sulfobetaine-3,4-ethylenedioxythiophene) (PSBEDOT), which could be facilely synthesized by electropolymerization in aqueous solution. The PSBEDOT surface exhibits excellent conductivity, stability and switchable antifouling/antimicrobial properties. It shows great promise for applications in organic bioelectronics. This new material may significantly increase the performance and service life, minimize the foreign body reaction, improve the biocompatibility and reduce the infection of bio-electronic devices.

## Abstract 2

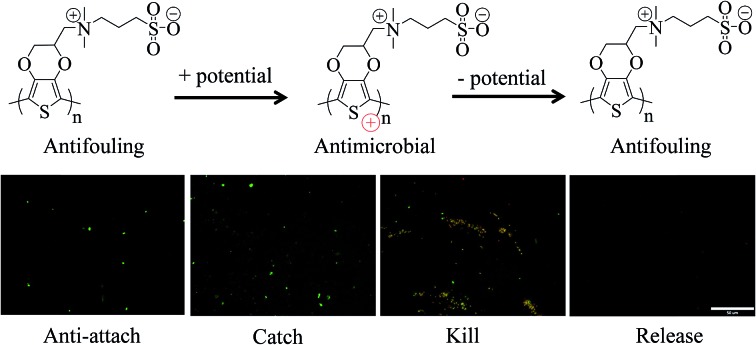
A multifunctional zwitterionic PSBEDOT material, which can switch between antifouling and antimicrobial states by controlling the potential of the surface, is synthesized.

## Introduction

Conjugated polymers (CPs) hold great promise for next-generation bioelectronics,^1^,^2^ because of their good compatibility with biological systems, design flexibility, ease of fabrication and relatively low costs. Previous studies found that CPs could improve the communication between electrochemical devices and biological systems initially;^3^–^5^ however, CPs such as polyacetylene,^6^ polyaniline (PANi),^7^ polypyrrole (PPy),^8^ polythiophene (PTh) and poly(3,4-thylenedioxythiophene) (PEDOT)^9^ were not originally designed for complex biological applications. When these polymers are used in biological systems, one major challenge is to maintain a “clean” and biocompatible biotic–abiotic interface to minimize foreign body reactions, reduce infections and prolong the service life of the device, while maintaining the material’s conductivity, stability and functionalities. Conventional CPs consist of hydrophobic or charged side chains. Biomolecules, mammalian cells and bacteria tend to attach to hydrophobic or charged surfaces. The adsorption of biomolecules and attachment of unwanted cells will reduce the sensitivity or lead to the failure of embedded devices.^10^,^11^ To increase the biocompatibility, PTh,^12^ PANi^13^ and PPy^14^ hydrogels have been developed to combine the electrical properties of CPs with the properties of hydrogels.^15^ To gain biocompatibility, CPs are blended or physically crosslinked with biocompatible and non-conducting polymers. For example, polyethylene glycol (PEG) was used to crosslink PANi for glucose sensing.^16^ However, non-conducting components compromise the electrochemical properties of the CPs.^17^ Furthermore, the non-conducting components of current conducting hydrogels are not effective enough to prevent long-term biofouling and foreign body responses. Previous studies discovered that zwitterionic polymers could effectively resist nonspecific protein adsorption and cell attachment.^18^–^20^ In our previous study, an integrated poly(carboxybetaine thiophene) (PCBTh) with both conducting and antifouling properties was developed.^21^ Due to the loosely packed polymer networks in the hydrogel, the electron conductivity needs to be further improved to meet the requirements of applications that demand high electron/current transport properties.

Herein, we have designed and synthesized a novel sulfobetaine-functionalized CP platform PSBEDOT, using poly(3,4-ethylenedioxythiophene) (PEDOT) as the conducting backbone due to its exceptional conductivity,^22^ low oxidation potential,^23^ relatively high chemical and thermal stability,^24^ and optical properties.^25^ SBEDOT monomers were polymerized on electrodes to form a densely packed film through electropolymerization in 100% aqueous solution. The PSBEDOT surfaces were designed to have electro-switchable antimicrobial/antifouling properties and excellent electrical conducting properties, to minimize infection, increase biocompatibility and improve the performance of bioelectronics. The conductivity, stability and antifouling properties against both proteins and cells, and the antimicrobial properties of the PSBEDOT surface, were systematically investigated during this work.

## Results and discussion

**Scheme 1:**
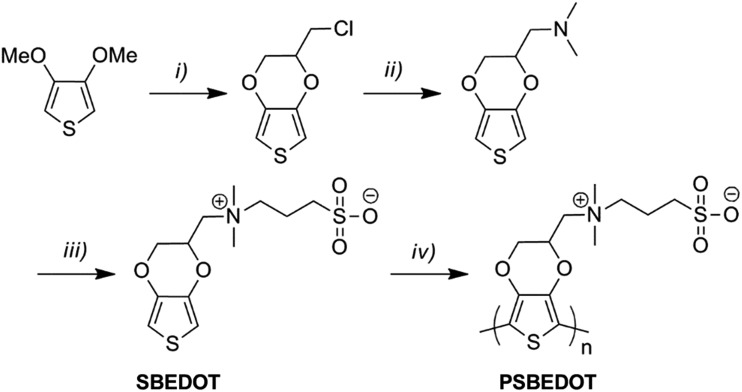
Synthetic route of PSBEDOT. Reaction conditions: (i) 3-chloropropane-1,2-diol, *p*-toluenesulfonic acid, toluene; (ii) dimethylamine, water, acetonitrile; (iii) 1,3-propanesultone, tetrahydrofuran; (iv) electropolymerization in aqueous solution (see ESI†).

As shown in Scheme 1, SBEDOT was synthesized using a three-step method. EDOT-Cl was firstly synthesized using reported procedures.^26^ A straightforward amination of EDOT-Cl, followed by quaternization with 1,3-propanesultone, produced the target zwitterionic compound, SBEDOT, with a good overall yield (see ESI†). The pure product was characterized using ^1^H and ^13^C nuclear magnetic resonance (NMR) spectroscopy (see ESI†). We used electropolymerization methods to prepare the CP surface, since they provide precise control of the polymer film growth on the electrode surfaces by simply adjusting the potential/current and reaction time.^27^ Both cyclic voltammetry (CV) and galvanostatic (GS) methods were used to polymerize SBEDOT on various substrates in an aqueous solution containing 60 mM monomer and 100 mM LiClO_4_ as the electrolyte. Zwitterionic PSBEDOT was successfully coated on both an indium tin oxide coated polyethylene terephthalate (ITO-PET) substrate and gold coated SPR sensor chips. The surfaces prepared using the GS method showed much better homogeneity than those generated from the CV method. It was noticed that, during the GS electropolymerization process, the working potential decreases smoothly with the increase in reaction time, indicating a decrease of the overall impedance and reflecting the excellent electrical conductivity of the deposited PSBEDOT films. EDOT monomers were also polymerized using a similar method on the same substrates and the PEDOT surface was used as a control throughout this study. It should be pointed out that much effort has been devoted to developing flexible, stable and biocompatible CP-based bioelectronic devices, but the conventional monomers are either poorly water-soluble or require the addition of surfactants to improve the aqueous processability.^28^ One major advantage of the SBEDOT monomer is that it is highly water-soluble and can be directly polymerized in aqueous solution without using organic solvent or surfactants, which significantly facilitates its future applications *in vivo*. The successful film deposition of PSBEDOT might be a result of the high polymerization rate, high molecular weight and anti-electrolyte effect of PSBEDOT polymers. A similar phenomenon was also observed by Dr Yu and co-workers.^28^

To confirm the successful surface deposition of the material, the detailed chemical composition of the PSBEDOT surface was analyzed with X-ray photoelectron spectroscopy (XPS). The peak areas, line shapes and intensities of the C 1s, O 1s, N 1s and S 2p high resolution spectra were monitored. In the survey spectrum of PSBEDOT (Fig. S5A†), the presence of N and a doublet of S, which were not present in PEDOT, indicated that PSBEDOT was successfully deposited onto the substrate. The atomic ratios were also in agreement with the molecular composition. The detailed high-resolution spectrum of S 2p shows that two types of S coexisted with nearly equivalent peak intensities, which confirmed the presence of immobilized PSBEDOT homopolymer with equal amounts of sulfur atoms in both the thiophene rings and ionic sulfonate side chains.

**Fig. 1:**
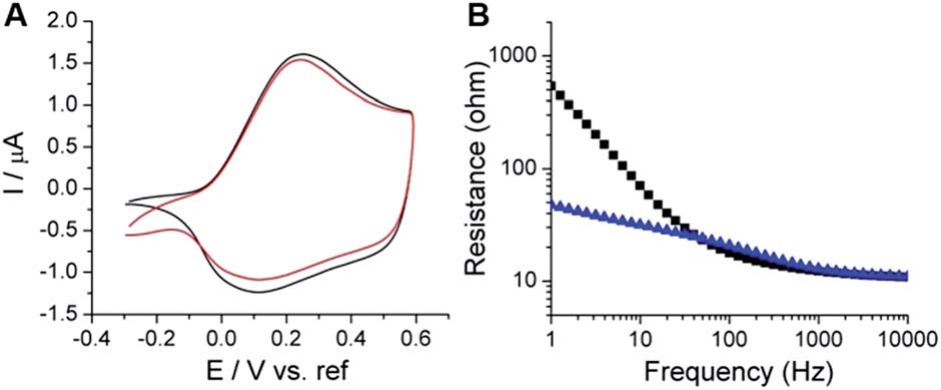
Electrochemical characterization of PSBEDOT coated substrates. (A) Comparison of the cyclic voltammograms of PSBEDOT on gold from the first cycle (black) and after 500 cycles (red) of the applied potential, (B) electrochemical impedance spectra (Bode plots) of the bare gold substrate (black squares) and PSBEDOT coated gold substrate (blue triangles).

To deliver/detect low electrical signals, both high electrochemical stability and low interfacial impedance are required for bioelectronics. CV and electrochemical impedance spectroscopy (EIS) methods were used to analyze the electrochemical properties of the coated films. The PSBEDOT films showed good stability, with a slight decrease of electro-activity after CV sweeping for 500 cycles from –0.3 to 0.6 V *vs.* a Hg/HgCl_2_ electrode (Fig. 1A). To determine the interfacial impedance of the PSBEDOT film, EIS was performed on both coated and uncoated substrates. The impedance of the PSBEDOT-coated substrate was about 10 times lower than the uncoated gold at low frequencies (Fig. 1B), which is comparable to that of PEDOT and suggests that a densely packed polymer layer was formed. Our result indicates that the PSBEDOT can significantly decrease the interfacial impedance of the gold electrode, which is highly desired and may significantly improve the signal collection and charge delivery of bioelectronics.^29^ It should be noted that dopants may affect the conductivity of the conjugated polymer films. To increase the conductivity of PEDOT, the polymer is usually doped with strongly acidic polystyrene sulfonate (PSS), which may potentially cause degradation of the adjacent non-noble metals/polymers or trigger inflammation. Since PSBEDOT was designed for biological applications, no leachable or acidic dopants were added. The electropolymerization of SBEDOT was conducted using lithium perchlorate as an electrolyte. The perchlorate anion was incorporated into the film as a dopant. To remove perchlorate, all surfaces are equilibrated in PBS solution, so perchlorate can be exchanged with other anions in the solution.

**Fig. 2:**
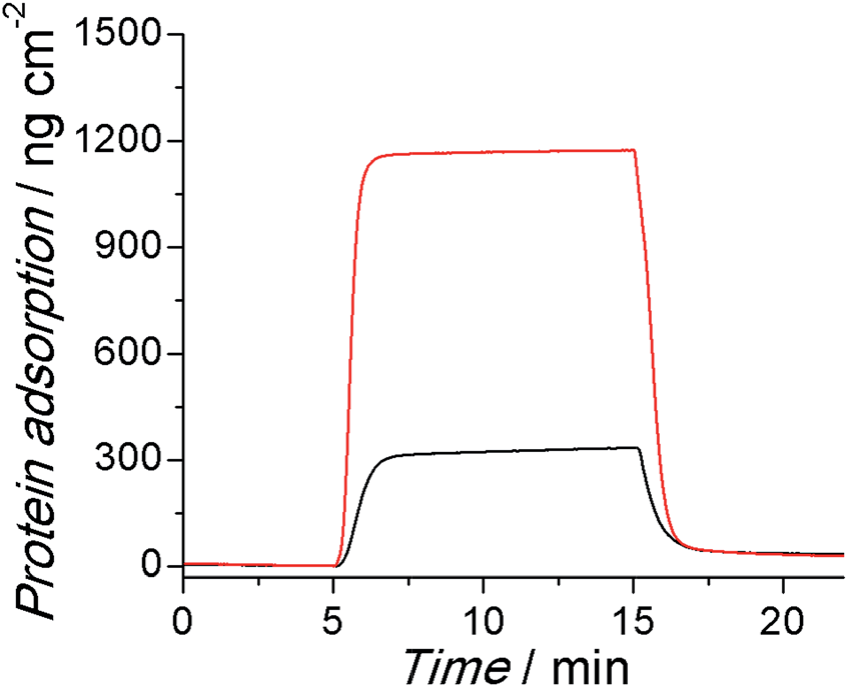
Representative SPR sensorgrams of PSBEDOT coated sensor chips, showing the low protein adsorption from 100% human blood plasma (red) and 30% human blood serum (black).

The adsorption of protein onto the surface of submerged objects in biological systems is one of the root causes of many biofouling phenomena, which eventually lead to the failure of bioelectronics. A four-channel surface plasmon resonance (SPR) sensor was used to evaluate the antifouling properties of the PSBEDOT coated gold chips using 100% human blood plasma and 30% human blood serum, which are two of the most complex protein solutions. As shown in Fig. 2, the PSBEDOT coated gold surface can effectively resist protein adsorption from both 100% human blood plasma and 30% human blood serum. The protein adsorption on the surface was calculated from the SPR wavelength shift before the protein injection and after the buffer wash. The adsorption amounts are about 28 ng cm^–2^ for plasma and 33 ng cm^–2^ for serum, which are slightly higher than those of non-conducting zwitterionic polymer brush surfaces, such as poly(carboxybetaine methacrylate)^30^ and its derivatives.^18^,^20^ For coatings, the packing density and surface roughness are two important factors for their antifouling properties. Compared to polymer brushes generated from atom transfer radical polymerization (ATRP), polymer films obtained from electrochemical polymerization may not be densely packed and well oriented, so this may increase the specific surface area of the substrate. Therefore, it is possible that the polymer architecture and high surface area resulting from electrochemical polymerization slightly compromise the antifouling performance; however, from the aspect of application, electropolymerized surfaces are easier to prepare and allow for more flexible control of the film thickness than polymer brush-based surfaces.

**Fig. 3:**
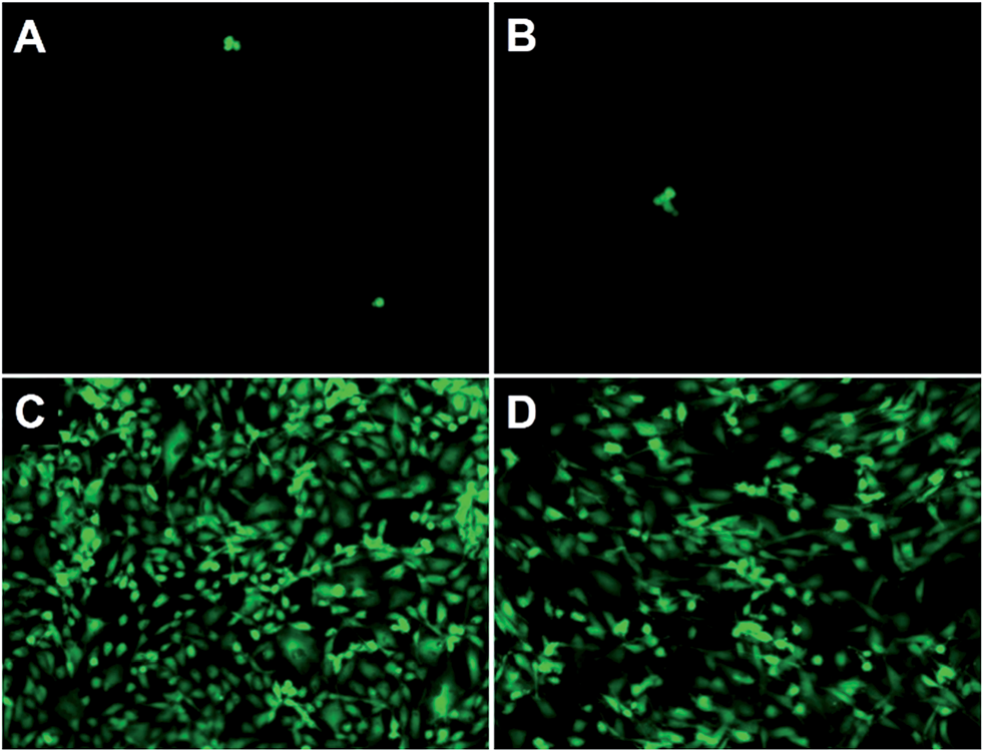
Cell adhesion tests of PSBEDOT-coated gold substrates incubated with (A) BAECs and (B) NIH 3T3 fibroblast cells, and the PEDOT-coated gold substrates incubated with (C) BAECs and (D) NIH 3T3 fibroblast cells for 24 hours.

To further evaluate the antifouling properties of the PSBEDOT surfaces, cell attachment studies were performed using both bovine aorta endothelial cells (BAECs) and mouse NIH 3T3 fibroblast cells. PSBEDOT and PEDOT surfaces were prepared from GS electropolymerization of SBEDOT and EDOT monomers on both ITO-PET surfaces and gold-coated SPR sensor chips. All cells were incubated with the substrates at 37 °C for 24 hours before imaging. For the PSBEDOT coated ITO-PET, a significant difference was observed between the coated and uncoated regions across the coating edge. A large amount of cells was found on the uncoated area of ITO-PET, while very few cells were found on the PSBEDOT-coated site (Fig. S8†). Both PSBEDOT and PEDOT were also coated on gold SPR substrates. Nearly a full coverage of BAECs and NIH 3T3 fibroblast cells was seen on the PEDOT surfaces, while there was almost no cell attachment on the PSBEDOT surfaces (Fig. 3). The densities of the adhered BAECs and NIH 3T3 fibroblast cells on the PSBEDOT surfaces were 0.7% and 0.9% of that on the PEDOT surfaces (Table S1†). These results demonstrate that the PSBEDOT surface highly resists nonspecific cell attachment.

**Fig. 4:**
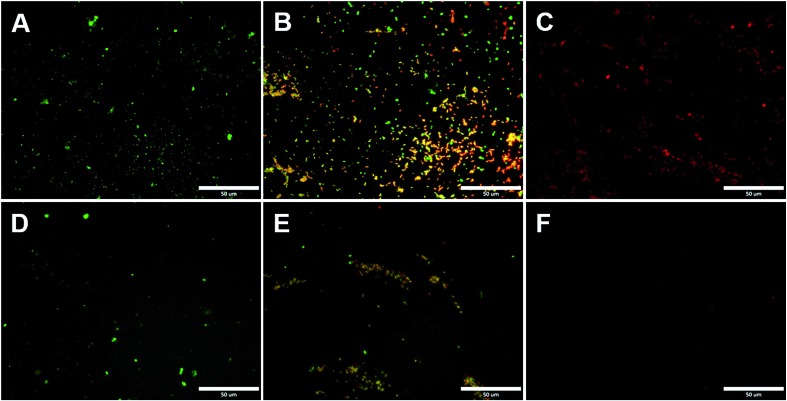
Representative fluorescence microscopy images of the bacterial adhesion, antimicrobial and release studies on PSBEDOT and control surfaces. Attached *E. coli* K12 from a suspension with 10^9^ cells per mL on gold (A) and oxidized PSBEDOT (D); the viability of the attached *E. coli* K12 on gold (B) and oxidized PSBEDOT (E) after subjection to 0.6 V for 1 hour; and the remaining *E. coli* K12 on gold (C) and oxidized PSBEDOT (F) after subjection to 0 V for 1 hour. In the viability study, bacterial cells were stained using a LIVE/DEAD BacLight Bacterial Viability assay kit. Cells with a damaged cytoplasm membrane are in yellow and red, and cells with an intact cytoplasm membrane are in green.

**Fig. 5:**
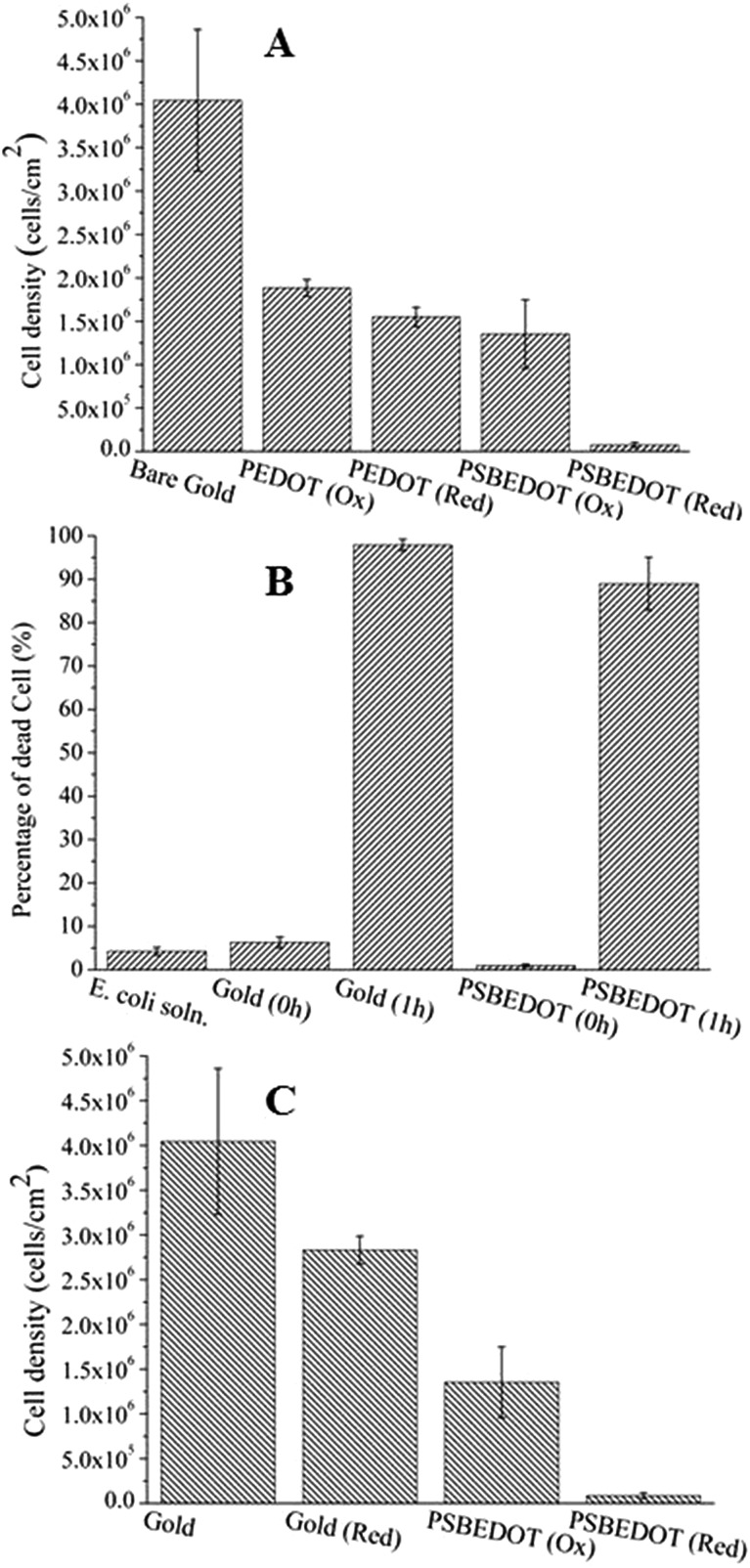
Quantitative bacterial adhesion, antimicrobial and release studies on PSBEDOT and control surfaces. (A) Attachment of *E. coli* K12 from a suspension with 10^9^ cells per mL on oxidized (Ox) PSBEDOT, reduced (Red) PSBEDOT and control surfaces; (B) bactericidal activity results of PSBEDOT and the control surface against *E. coli* K12 after subjection to 0.6 V for 1 hour; and (C) detachment of *E. coli* K12 from oxidized PSBEDOT and gold after subjection to 0 V for 1 hour.

One major challenge of implantable devices/materials is the surgical infection. To prevent infections, antifouling and antimicrobial strategies are commonly used. Due to the unique structure of the zwitterionic conjugated PSBEDOT, we expect that PSBEDOT can switch between antifouling and antimicrobial states under different potentials. In the oxidized state, the PSBEDOT backbone is positively charged and the overall polymer becomes cationic. In the reduced state, the PSBEDOT backbone is neutral, so the polymer remains in its zwitterionic state. To evaluate PSBEDOT's potential to minimize infections, bacterial adhesion, antimicrobial and releasing studies on the PSBEDOT surfaces were conducted using *E. coli* K12 as a model species. Before the attachment study, the PSBEDOT substrates were equilibrated at 0.6 or 0 V in PBS for 30 minutes to generate oxidized and reduced PSBEDOT surfaces respectively. The bacterial attachment study (Fig. 4 and 5A) showed that the reduced PSBEDOT surfaces were highly resistant to the attachment of *E. coli* K12 at a very high concentration (10^9^ cells per mL). After 1 hour, the cell density on the reduced PSBEDOT surface was less than 1.9% of that on the gold surface. The attachment of *E. coli* K12 on the oxidized PSBEDOT surface increased to 33.4% relative to the gold surface. The densities of the attached *E. coli* K12 cells on both the oxidized and reduced PEDOT surfaces were high (46.6% and 38.4% relative to the gold surface). The excellent antifouling properties of being able to resist bacterial attachment on the reduced PSBEDOT surface are due to its strong hydration properties and a similar phenomenon was also observed for poly(sulfobetaine methacrylate) (PSBMA) polymer brush surfaces.^30^ Previous studies demonstrated that there was a direct correlation between bacterial attachment and biofilm development.^30^,^31^ The lower bacterial attachment on both the oxidized and reduced PSBEDOT surfaces can potentially minimize infections.

To confirm that oxidized PSBEDOT can kill attached bacterial cells, PSBEDOT substrates with attached cells were submerged in PBS and a 0.6 V potential was applied for 1 hour. Before and after applying the potential, the viability of the attached bacterial cells was analyzed with LIVE/DEAD® Cell Viability Assays using a fluorescence microscope. The results in Fig. 5B show that the PSBEDOT surfaces caused membrane damage to 89% of the *E. coli* in one hour and the gold substrate killed >97.9% of the attached cells. In solution, over 95.8% of *E. coli* K12 cells were still viable after one hour. One advantage of CP surfaces is that the surface potential can be actively controlled. By applying a lower potential (0 V), the oxidized cationic PSBDEOT surface can switch to a reduced zwitterionic surface. Due to the repulsive force generated by strong hydration of the zwitterionic side chains and the disappearance of the attractive force between the negatively charged bacteria and positively charged PSBDEOT surfaces, the killed bacterial cells can be released. To confirm the hypothesis, a bacterial cell releasing experiment was conducted using PSBEDOT and gold surfaces that carried killed bacterial cells from the antimicrobial study. As shown in Fig. 5C, 96.7% of *E. coli* K12 cells on the PSBDEOT surface were released within 1 hour under the static conditions after the potential was decreased to 0 V from 0.6 V, while only 30% of the cells on the gold surface were released. The final cell density on PSBEDOT was less than 3% of that on the gold substrate. It should be noted that the release of killed bacterial cells is critical for implanted materials, since the attached dead cells may cause chronic inflammation and lead to the failure of implanted materials/devices. Previously several switchable antifouling/antimicrobial materials have been reported.^18^,^20^,^32^ These zwitterionic polymers can undergo ring formation to become cationic under low pH conditions (pH < 5) and can switch back to their zwitterionic state under neutral or basic conditions. In this work, the electrochemical approach allows for more rapid and active control of the state of the zwitterionic materials. Through this study, we have demonstrated that PSBEDOT surfaces could effectively resist cell attachment in their reduced state, kill the small amount of attached cells in their oxidized state and release the dead cells after switching back to the reduced state.

Numerous applications, ranging from the field of solid state technology^33^,^34^ to biomedical engineering,^33^,^35^,^36^ need to use high performance CPs as the key components that determine the function and properties of the devices, so the development of novel multifunctional CPs is of great importance. One of the most attractive features of CPs over traditional biomaterials is that they could allow electrical stimulation of the attached tissues and cells.^37^ It is expected that the novel PSBEDOT could be used to manipulate cell attachment through electrochemical control and also could serve as a protective coating to reduce protein adsorption and cell attachment thus prolonging the lifetime of implanted devices. Although there is much work to be done to fully understand and realize the potential of zwitterionic conjugated polymers, we believe this work will fundamentally advance the development of bioelectronics.

## Conclusions

In this work we designed and synthesized a novel antifouling and electroactive PSBEDOT material. Zwitterionic PSBEDOT can be facilely polymerized in aqueous solution through an electrochemical method. The PSBEDOT polymer films exhibit excellent electrochemical properties, low interfacial impedance, stability and switchable antifouling/antimicrobial properties. The interfacial impedance of PSBEDOT was less than 10% of that of bare gold at low frequency. It also showed superior antifouling properties against whole blood, mammalian cells and bacteria. The PSBEDOT surface can also be switched between cationic antimicrobial and zwitterionic antifouling states by applying different potentials. It can kill over 89% of attached cells in one hour at 0.6 V and release over 96.7% of the dead cells in one hour at 0 V under static conditions. It shows great promise for applications in bioelectronics. This new material may significantly increase the performance and service life, minimize the foreign body reaction, improve the biocompatibility and reduce the infection of bio-electronic devices.

## Supplementary Material

Supplementary information
