# Supplementary material for: Electroactive poly(sulfobetaine-3,4-ethylenedioxythiophene) (PSBEDOT) with controllable antifouling and antimicrobial properties
Source: Chem Sci. 2015 Dec 18;7(3):1976–81. doi: 10.1039/c5sc03887a (PMC5966908; doi:10.1039/c5sc03887a)
Supplement: Supplementary file 1 [file SC-007-C5SC03887A-s001.pdf]

Electronic Supplementary Material (ESI) for Chemical Science.
This journal is © The Royal Society of Chemistry 2015

Supplementary Information for

Electroactive Poly(sulfobetaine 3,4-ethylenedioxythiophene) (PSBEDOT) with Controllable Antifouling and Antimicrobial Properties

Bin Cao,[a] Chen-Jung Lee,[a] Zhipeng Zeng,[a] Fang Cheng,[b] Fujian Xu,[c] Hongbo Cong,[a] and Gang Cheng*[a]*

[a] Department of Chemical and Biomolecular Engineering, University of Akron, Akron, Ohio 44325, USA Fax: (+01) 330-972-7250
Dr. Gang Cheng E-mail: gc@uakron.edu
Homepage: http://gozips.uakron.edu/~gc/index.html
Dr. Hongbo Cong E-mail: hcong@uakron.edu
[b] School of Pharmaceutical Engineering, Dalian University of Technology, Dalian, Liaoning Province, 116024, China.
[c] Key Laboratory of Carbon Fiber and Functional Polymers (Beijing University of Chemical Technology), Ministry of Education, Beijing 100029 China and Beijing Laboratory of Biomedical Materials, Beijing University of Chemical Technology, Beijing 100029, China

Table of Contents

1. Chemicals and general instrumentation S2
2. Synthetic procedures S2
3. Electropolymerization of SBEDOT S5
4. X-Ray photoelectron spectroscopy (XPS) study S6
5. Electrochemical characterization of pSBEDOT S6
6. BAECs and NIH3T3 cell adhesion study S7
7. Protein adsorption study S8
8. Bacterial adhesion, antimicrobial and releasing study S9

Experimental methods

1. Chemicals and general instrumentation

3,4-Dimethoxythiophene was purchased from Matrix Scientific (Columbia, SC, USA). (±)-3-chloro-1,2-propanediol, toluene, p-Toluenesulfonic acid monohydrate, dimethylamine solution (40 wt. % in H_2O), acetonitrile, 1,3-propanesultone, anhydrous magnesium sulfate, anhydrous tetrahydrofuran(THF), chloroform, methanol, dichloromethane, ethyl acetate, phosphate-buffered saline(PBS) and fluorescein diacetate were purchased from Sigma-Aldrich (St. Louis, MO, USA). All chemicals were used as received without further purification. Bovine aorta endothelial cell (BAEC) and Mouse NIH 3T3 fibroblast cell were purchased from American Type Culture Collection (Manassas, MD, USA). Dulbecco's Modified Eagle's Medium (DMEM) was purchased from Life Technologies (Carlsbad, CA, USA). Water used in all experiments was purified using a Millipore Milli-Q Direct 8 Ultrapure Water system (Billerica, MA, USA). Electro-polymerization and other electrochemical characterizations were performed on a Solartron Modulab XM ECS test system or a Gamry Reference 600 potentiostat. XPS spectra were obtained from a PHI VersaProbe II Scanning XPS Microprobe. All NMR experiments were performed at 303.2 K unless stated otherwise and on Varian Mercury 300 MHz spectrometers.

2. Synthetic procedures

Chloromethyl-EDOT was first synthesized following a method published previously.[1] EDOT-dimethylamine was synthesized as a versatile intermediate, which was used to synthesize zwitterionic EDOT derivatives bearing carboxybetaine or sulfobetaine side chains.

2.1 Synthesis of EDOT-dimethylamine (EDOT-DMA)

Chloromethyl-EDOT (3.8 g, 20 mmol) was added to a solution of dimethylamine (40 wt. % in H_2O) (22.5 mL, 200 mmol) and acetonitrile (22.5 mL). The mixture was sealed in a schlenk flask and heat at 80 ^oC for 2 days. Another 22.5 mL of dimethylamine (40 wt. % in H2O) was added after it cooled down. Then the solution was heated at 80 ^oC for another 36 hours. After the solution cooled to room temperature, it was concentrated with a rotary evaporator, extracted with ether, dried with MgSO4. Product was purified with silica gel column chromatography

(MeOH/CH2Cl2/ethyl acetate, 1/10/10 (v/v/v)). Pure product was obtained as a light yellowish liquid (Yield: 65 %). ^{1}H NMR (300 MHz, CDCl3) δ 6.30-6.36 (m, 2H), 4.20-4.30 (m, 2H), 3.90-3.97 (m, 1H), 2.41-2.64 (m, 2H), 2.31 (s, 6H). ^{13}C NMR (75 MHz, CDCl3) δ 141.84, 99.90, 99.55, 71.95, 67.52, 59.65 (one carbon not seen due to overlapping signal)

2.2 Synthesis of SBEDOT

1,3-Propanesultone (1.46 g, 12 mmol) was slowly added into a solution of EDOT-dimethylamine (2.0 g, 10 mmol) in 50 mL of anhydrous THF. The mixture was heated at 55 ^{o}C for 36 hours under a positive nitrogen flow. After filtration, washed with THF and vacuum dried, pure product was obtained as a white powder (Yield: 82%). ^{1}H NMR (300 MHz, D2O) δ 6.58-6.65 (m, 2H), 5.00 (m, 1H), 4.14-4.31 (m, 2H), 3.62-3.85 (m, 4H), 3.27 (s, 3H), 3.25 (s, 3H), 2.98 (t, 2H, *J* = 7.2 Hz), 2.22 (m, 2H). ^{13}C NMR (75 MHz, D2O) δ 139.94, 138.45, 101.66, 101.00, 67.80, 65.90, 63.69, 62.55, 51.97, 51.69, 47.16

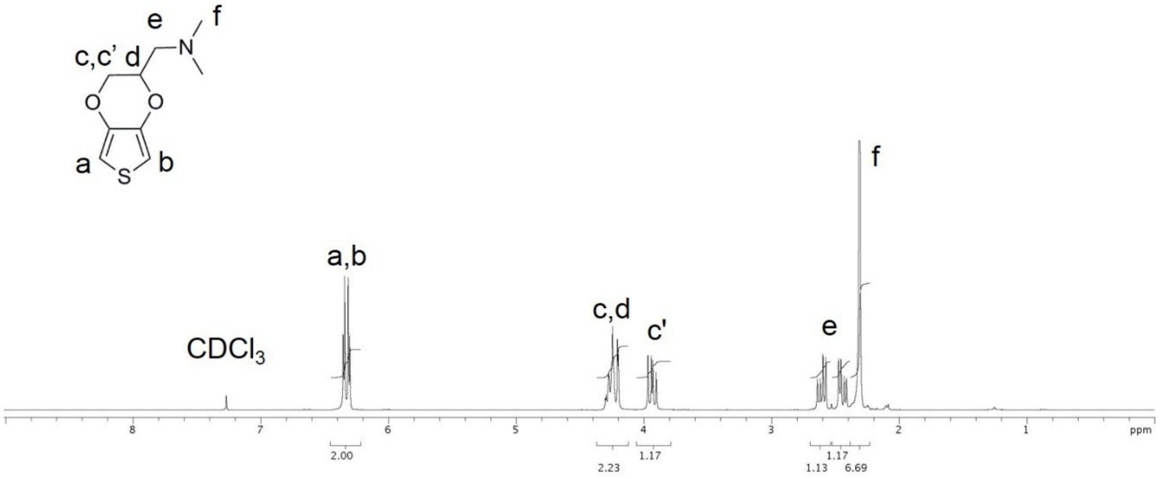

Figure S1. ^{1}H spectrum of EDOT-DMA

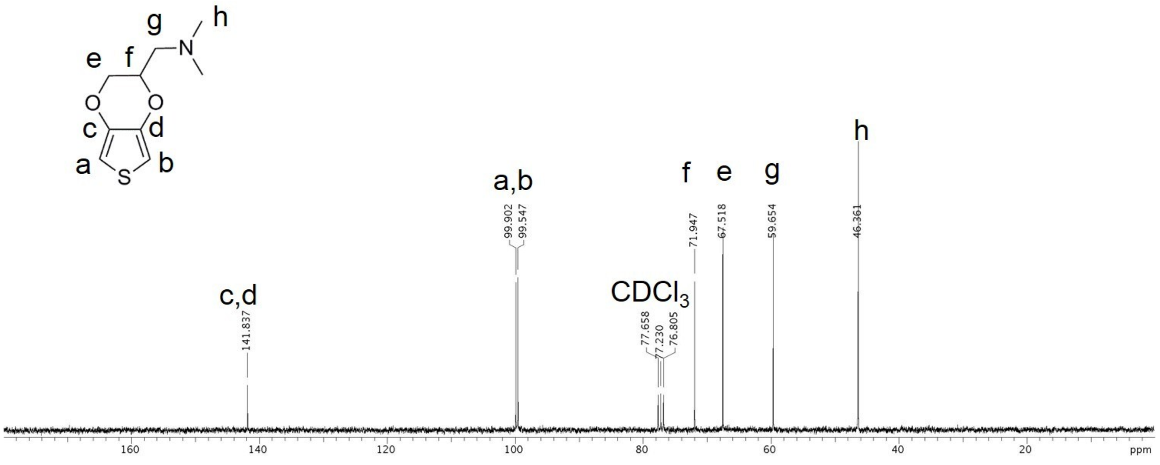

Figure S2. ^{13}C spectrum of EDOT-DMA

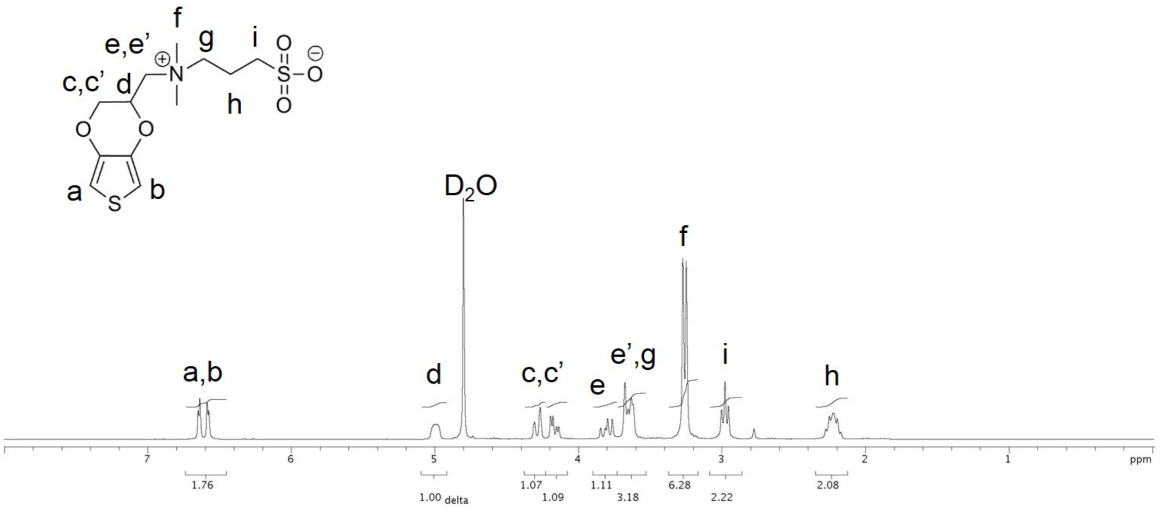

Figure S3. ^{1}H spectrum of SBEDOT

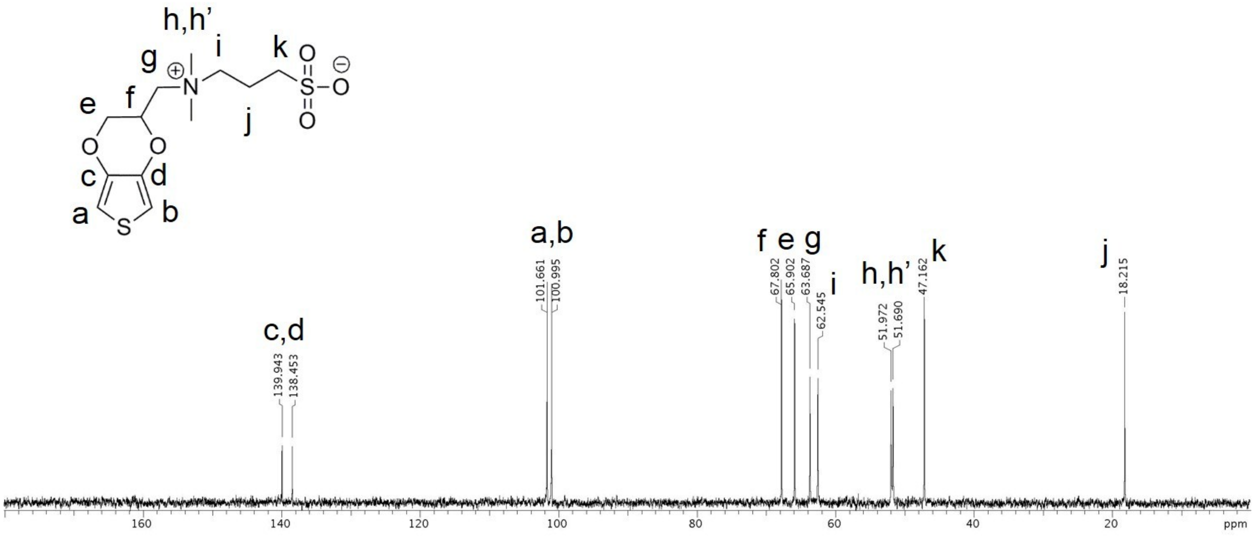

Figure S4. ^{13}C spectrum of SBEDOT

3. Electropolymerization of SBEDOT

Electropolymerization was performed on a Solartron Modulab XM ECS test system or a Gamry Reference 600 potentiostat equipped with a three-electrode electrochemical set-up, using a Pt electrode as the counter electrode and an $Hg/HgCl_2$ electrode (sat. KCl) as the reference electrode. One of the great advantages of this SBEDOT monomer is it could be directly polymerized in aqueous solution, which significantly facilitates its future applications *in vivo*. SBEDOT was polymerized on either ITO coated PET films or Gold coated SPR sensor chips (Figure s5), with cyclic voltammetry from -0.6 V to 1.3 V, or galvanostatic method at 0.1 mA/s, from an aqueous solution containing 60 mM monomer and 100 mM LiClO4 as electrolyte.

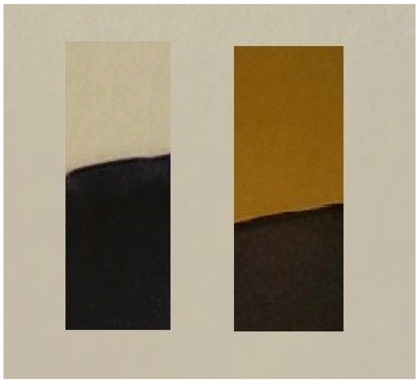

Figure S5. Optical images of PSBEDOT coating on ITO (left) and gold (right) substrates.

4. X-Ray photoelectron spectroscopy (XPS) study

XPS was also used to examine the composition profile of electropolymerized PSBEDOT film, using a PHI VersaProbe II Scanning XPS Microprobe. All data processing were performed using the software provided with the instrument. The PSBEDOT samples were run for both the survey and the high resolution spectra. The survey spectra of PEDOT sample was used for comparison. All data processing were performed using the software provided with the instrument. Peak areas, line shapes, and intensities of C 1s, O 1s, N 1s and S 2p high resolution spectra were monitored. As shown in Figure S6, The atomic ratios were in agreement with molecular compositions. From the S 2p high resolution spectra of PSBEDOT, two types of S were observed with equivalent peak intensity, indicating its elemental and chemical composition was exactly the same as expected.

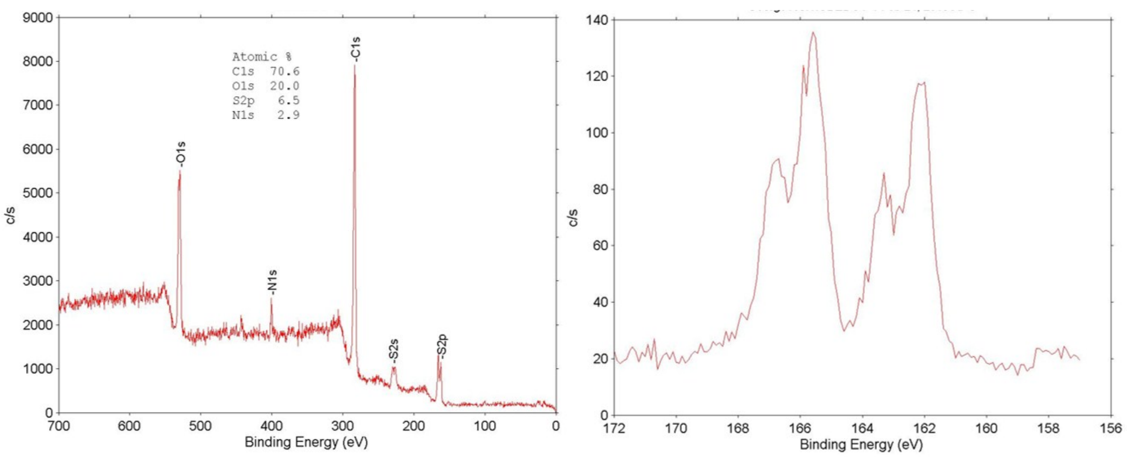

Figure S6. The XPS profiles of PSBEDOT coating. Survey spectrum (left), high-resolution spectrum of S 2p (right).

5. Electrochemical characterization of PSBEDOT

Electrochemical impedance spectroscopy (EIS) and Cyclic Voltammetry (CV) were performed in PBS using a Gamry Reference 600 potentiostat in PBS buffer. Stability measurement of PSBEDOT film was carried out with CV (-0.3 V to 0.6 V). Although PSBEDOT was hydrophilic, it showed excellent stability in aqueous solution, even after applying a potential sweep for over 500 cycles (Figure S7). The PSBEDOT films for measurements were coated on gold coated SPR sensor chips. For EIS, the frequencies were spaced from 10 kHz to 1 Hz with a

low amplitude voltage (~10 mV). Before EIS experiment, samples were equilibrated in PBS buffer for 10 minutes. Potentiodynamic study of PSBEDOT film was recorded with CV, from -0.8 V to 1.0 V, at different scan rates of 10, 20, 50, 100, 200 mv/s.

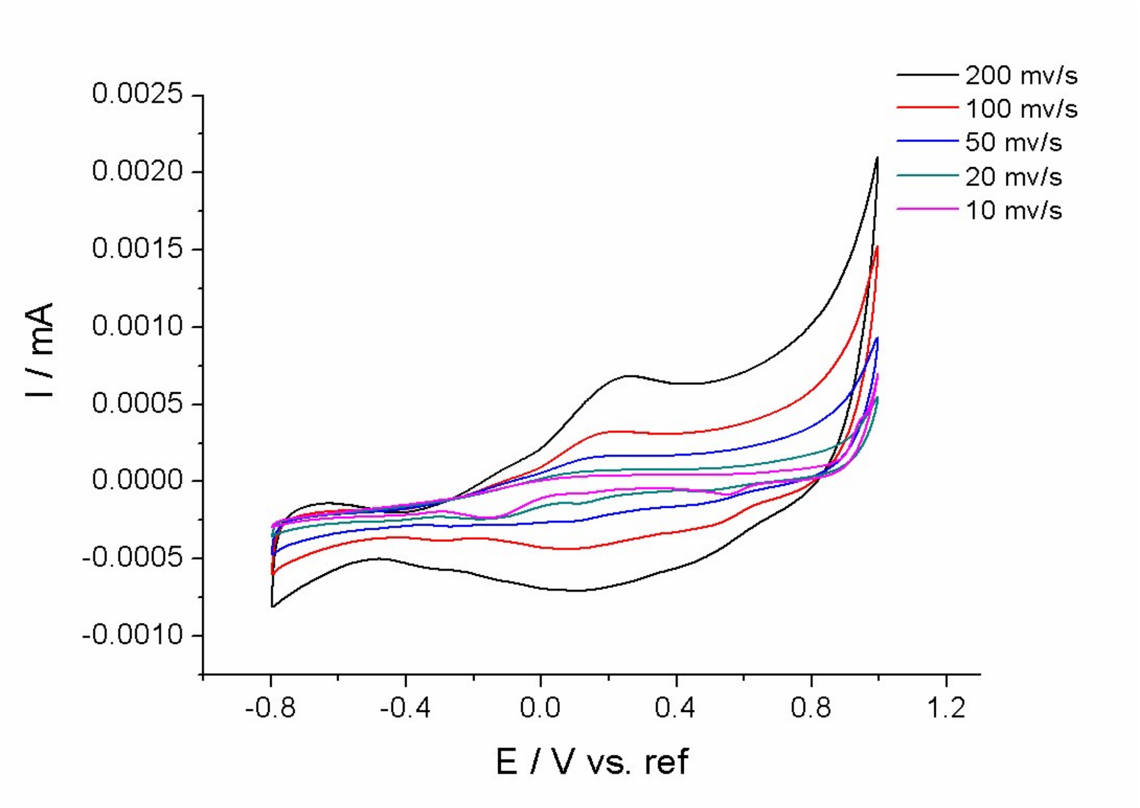

Figure S7. Cyclic voltammograms of PSBEDOT film at different scan rate.

6. BAEC and NIH-3T3 cell adhesion study

Bovine aorta endothelial cell (BAEC) and NIH3T3 were purchased from American Type Culture Collection (Manassas, MD, USA). Cell attachment study was carried out following a similar procedure to that used in a previous work. PSBEDOT and PEDOT was electro-deposited on ITO coated PET substrates, then equilibrated in DI-water for 24 hours and transferred to sterilized PBS. All samples were exposed under UV for half an hour before the cell adhesion experiment.

BAECs and NIH3T3 were separately seeded on different substrates at 10^5 cells/mL with DMEM medium consisting of DMEM, 10% fetal bovine serum (FBS), and 1% penicillin–streptomycin and kept in an incubator with 5% CO_2 at 37 ^{o}C for 24 hours. After the incubation, medium was

removed from the wells. After very gently rinsed with sterilized PBS, it was changed to the staining solution that prepared in sterilized PBS as follows. Fluorescein diacetate was dissolved at a concentration of 10 mg mL^{-1} in acetone, then 50 µL of the solution was diluted in 10 mL sterilized PBS and used for staining the cells. After incubated for 5 min with the staining solution, surface cell coverage and cell morphology was visualized and imaged with an Olympus IX81 fluorescence microscope (Olympus, Japan) equipped with a FITC filter at 4× or 10× magnification.

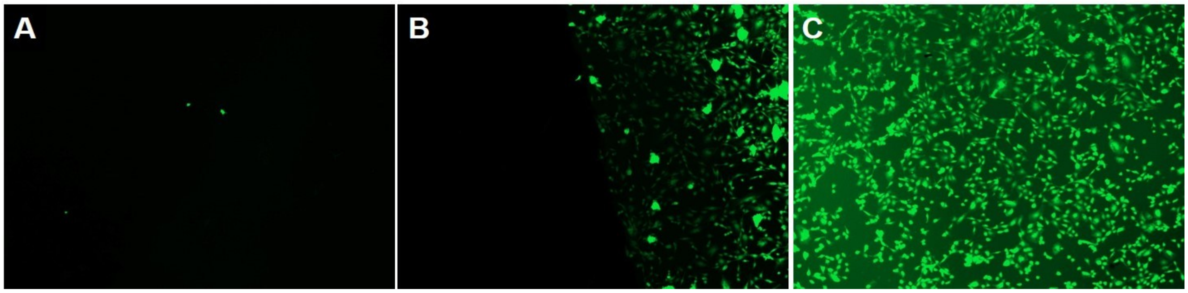

Figure S8. BAECs adhesion test with PSBEDOT coated ITO-PET. (A) PSBEDOT coated region, (B) Region across coating boundary and (C) uncoated region.

Table S1. Percentage of the attached cells on PSBEDOT surfaces relative to PEDOT coated surfaces (n = 3)

	BAECs on PSBEDOT	BAECs on PEDOT	NIH-3T3 on PSBEDOT	NIH-3T3 on PEDOT
% of cell attachment	0.7±0.2	100±3.6	0.9±0.4	100±11.2

7. Protein adsorption study

7.1 Protein adsorption study - SPR

A custom-built four-channel SPR sensor was used to measure protein adsorption on PSBEDOT surface. Firstly, PBS solution at a 50 µL min^{-1} flow rate was used to obtain a baseline signal. 100% human blood plasma and 30% diluted human blood serum were then injected into different channels for 10 minutes followed by a PBS wash to remove any loosely bound proteins. The amount of adsorbed proteins was calculated as the change in wavelength before and after protein injection.

7.2 FITC-labeled fibrinogen adsorption study - fluorescence microscopy

After equilibrated in PBS, the substrates was gently rinsed with DI-water and then transferred into a sterile 12-well plate. 4 mL of FITC-labelled fibrinogen (FITC-Fg) solution (0.1 mg/mL) was added into each well. All samples were immersed in the solution for 30 minutes to allow protein adsorption on substrate surfaces. To remove loosely adsorbed proteins on sample surfaces, all samples were gently rinsed with PBS. Protein adsorption on each substrate surface was visualized with an Olympus IX81 fluorescent microscopy (Olympus, Japan) with 4x objective lens through FITC filter at a fixed exposure time for all samples, so the different protein adsorption will lead to different fluorescent intensity on images. ImageJ software was used to quantify the fluorescent intensity of each sample. The results are shown in Figure S7.

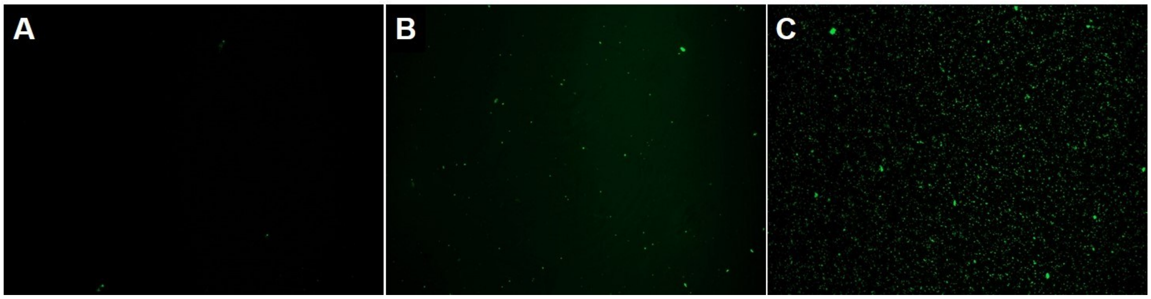

Figure S9. Protein (FITC-Fg) adsorption test on surfaces visualized under fluorescence microscope at the same excitation light intensity and exposure time. (A) PSBEDOT coated surface, (B) PEDOT coated surface, (C) bare gold sensor chip surface.

8. Bacterial adhesion, antimicrobial and releasing study

The method for evaluating the antibacterial efficiency of polymer surfaces was modified from a previously published method [2]. *E. coli* K12 was first cultured in separate pure cultures overnight at 37 °C on Luria-Bertani (LB) agar plates. One colony was used to inoculate 5 mL of LB medium (20 g/L). These initial cultures were incubated at 37 °C with shaking at 200 rpm for 12 hours. This culture was then used to inoculate a second culture in 25 mL of LB medium. When the second suspended culture reached an optical density of 0.8 at 600 nm, bacteria were collected by centrifugation at 8,000 x g for 10 min at 4 °C. Cell pellets were washed three times

with sterile PBS (pH 7.4) and subsequently suspended in PBS to get a final concentration of 10^9 cells/mL.

8.1 Bacterial attachment study

Before the bacterial attachment study, PSBEDOT coated Au substrates was equilibrated under 0.6 V and 0 V in PBS for 20 minutes to obtain surface at the oxidized state and reduced state respectively. A 0.1 mL suspension of *E. coli* at a concentration of10^9 cells/mL was pipetted onto each PSBEDOT coated Au substrate and then covered with a glass cover slip. The sample was incubated at room temperature for 1 hour. The cover slide was removed and the sample was rinsed in 50 mL of PBS. Then, the sample in PBS was stained with 1 mL of water containing 20 μM of red fluorescent nucleic acid stain propidium iodide (Life Technologies, Carlsbad, CA). The number of cells was determined with a CCD-CoolSNAP camera (Roper scientific, Inc., USA) mounted on Olympus IX81 fluorescent microscopy (Olympus, Japan) with 40x objective lens through FITC filter. Three separate samples were analyzed for each coating.

8.2 Antimicrobial study

After the cell attachment study, same PSBEDOT substrates with attached cells were transferred to PBS solution and 0.6 V potential was applied for 1 h. The sample was rinsed in 50 mL of PBS. Then, the sample in PBS was stained with 1 mL of water containing 20μM of red fluorescent nucleic acid stain propidium iodide and 3.34 μM green fluorescent nucleic acid stain SYTO9 (Life Technologies, Carlsbad, CA). The number of live and dead cells was determined with a CCD-CoolSNAP camera (Roper scientific, Inc., USA) mounted on Olympus IX81 fluorescent microscopy (Olympus, Japan) with 40x objective lens through FITC filter and Texas Red filter at a fixed exposure time for all samples. Three separate samples were analyzed for each coating.

8.3 Bacterial release study

After the antimicrobial study, same PSBEDOT substrates with attached cells were transferred to PBS solution and 0 V potential was applied for 1 h. The sample was rinsed in 50 mL of PBS. The number of live and dead cells was determined with a CCD-CoolSNAP camera (Roper scientific, Inc., USA) mounted on Olympus IX81 fluorescent microscopy (Olympus,

Japan) with 40x objective lens through FITC filter and Texas Red filter at a fixed exposure time for all samples. Three separate samples were analyzed for each coating.

1. J. L. Segura, R. Gómez, R. Blanco, E. Reinold and P. Bäuerle, *Chem Mater*, 2006, **18**, 2834-2847.
2. J. C. Tiller, C. J. Liao, K. Lewis and A. M. Klibanov, *Proceedings of the National Academy of Sciences of the United States of America*, 2001, **98**, 5981-5985.
